# Variability in the Insect and Plant Adhesins, *Mad1* and *Mad2*, within the Fungal Genus *Metarhizium* Suggest Plant Adaptation as an Evolutionary Force

**DOI:** 10.1371/journal.pone.0059357

**Published:** 2013-03-13

**Authors:** Michael Wyrebek, Michael J. Bidochka

**Affiliations:** Department of Biological Sciences, Brock University, St. Catharines, Ontario, Canada; University of Ottawa, Canada

## Abstract

Several species of the insect pathogenic fungus *Metarhizium* are associated with certain plant types and genome analyses suggested a bifunctional lifestyle; as an insect pathogen and as a plant symbiont. Here we wanted to explore whether there was more variation in genes devoted to plant association (*Mad2*) or to insect association (*Mad1*) overall in the genus *Metarhizium*. Greater divergence within the genus *Metarhizium* in one of these genes may provide evidence for whether host insect or plant is a driving force in adaptation and evolution in the genus *Metarhizium*. We compared differences in variation in the insect adhesin gene, *Mad1*, which enables attachment to insect cuticle, and the plant adhesin gene, *Mad2*, which enables attachment to plants. Overall variation for the *Mad1* promoter region (7.1%), *Mad1* open reading frame (6.7%), and *Mad2* open reading frame (7.4%) were similar, while it was higher in the *Mad2* promoter region (9.9%). Analysis of the transcriptional elements within the *Mad2* promoter region revealed variable STRE, PDS, degenerative TATA box, and TATA box-like regions, while this level of variation was not found for *Mad1*. Sequences were also phylogenetically compared to EF-1α, which is used for species identification, in 14 isolates representing 7 different species in the genus *Metarhizium*. Phylogenetic analysis demonstrated that the *Mad2* phylogeny is more congruent with 5′ EF-1α than *Mad1*. This would suggest that *Mad2* has diverged among *Metarhizium* lineages, contributing to clade- and species-specific variation, while it appears that *Mad1* has been largely conserved. While other abiotic and biotic factors cannot be excluded in contributing to divergence, these results suggest that plant relationships, rather than insect host, have been a major driving factor in the divergence of the genus *Metarhizium*.

## Introduction

Species within the genus *Metarhizium* are insect pathogenic fungi with a broad range of insect hosts. The genus was recently divided into several separate species based on a multilocus phylogeny [1]. The EF-1α sequence was found to be diagnostic for species identification. The population biology (and now species association) of *Metarhizium* had been assumed to be influenced primarily by host insect taxa [2]–[8]. That is, different species of *Metarhizium* were associated with different insect species. However, an association between *Metarhizium* species and habitat and/or plant types has been observed [9], [10]. This represents a significant paradigm shift, in that it demonstrated that habitat/plant selection, not host insect selection, influenced the population structure of *Metarhizium*. In addition, *M. robertsii* has been shown to be rhizosphere competent [11]–[13], further supported by research demonstrating *M. robertsii* is an endophyte [14].

*Metarhizium* is phylogenetically related to the fungal grass endosymbionts *Claviceps* and *Epichloë* [15]. Genomic analyses also indicated that *Metarhizium* spp. are more closely related to endophytes and plant pathogens than to animal pathogens, suggesting that *Metarhizium* evolved from fungi that are plant associates [16].

Two adhesin genes have been identified that are specifically involved with insect pathogenesis and plant association, *Metarhizium* adhesin-like protein 1 (*Mad1*) and *Metarhizium* adhesin-like protein 2 (*Mad2*), respectively [17]. The MAD1 adhesin allows *Metarhizium* to adhere to insect cuticle, while the MAD2 adhesin enables attachment to plants, and were expressed differentially on their respective hosts [17]. Both proteins contain a middle region (domain B) that contains Thr-rich tandem repeats.

We propose three possible models of evolution within genus *Metarhizium*: (1) insect host has caused divergence among species; (2) plant host has caused divergence among species; (3) other abiotic or biotic factors caused the divergence and evolution among *Metarhizium* species. In this study, we explored the genetic differences in 14 *Metarhizium* isolates, representing 7 different species, through sequence analysis (open reading frames and promoter regions) of the *Mad1* insect adhesin and *Mad2* plant adhesin genes. Sequences were also compared to the EF-1α gene, which allows for species identification [1], in order to infer evolutionary relationships.

## Results

### *Mad1* variability

Inter-isolate, interspecies, and intraspecies variation were calculated for the open reading frame and promoter regions through pairwise nucleotide comparisons. The greatest inter-isolate divergence within the open reading frame of *Mad1* was 14.2% found between isolates ARSEF 7486 (*M. acridum*) and ARSEF 6238 (*M. guizhouense*). However, when considering the average inter-isolate variation between species, the greatest interspecies divergence was 12.3% between *M. acridum* and *M. majus*, with the least divergence between *M. robertsii* and *M. brunneum* (2.9%). The overall average interspecies variation for the *Mad1* open reading frame for all *Metarhizium* species examined was 6.7%. The average interspecies variation for the promoter region was 7.1%. For the open reading frame, the intraspecies variation was low in *M. robertsii* (0.2%) and *M. brunneum* (0.3%), while it was relatively higher for *M. guizhouense* (3.9%). Similarly, in the promoter region, intraspecies variation was low in *M. robertsii* (0.1%) and *M. brunneum* (0.1%), and higher for *M. guizhouense* (3.9%). The average estimated nonsynonymous/synonymous substitution rate ratio (dN/dS) for *Mad1* was calculated at 0.20.

Initial analysis of the MAD1 proteins showed that *M. robertsii* isolates had a conserved protein length at 717 amino acids. The MAD1 protein for ARSEF 6238 (*M. guizhouense*) was also 717 a.a., while the Ontario isolates of *M. guizhouense* had proteins that contained 706 a.a. The MAD1 protein for *M. brunneum* isolates was 711 a.a., while the *M. pingshaense* MAD1 was 704 a.a. Overall, *M. acridum* had the longest MAD1 protein at 723 a.a., including an insertion of 11 amino acids within domain B, which contained Thr-rich tandem repeats. These 11 extra amino acids provided *M. acridum* with eight tandem repeats, while all other species contained six. *M. acridum* also possessed a variable region in the N-terminal ligand binding region of the protein, while this region was mostly conserved among other species.

### *Mad2* variability

The greatest inter-isolate divergence within the open reading frame for *Mad2* was 15.9% found between isolates ARSEF 7486 (*M. acridum*) and HKB1-1b (*M. robertsii*). Similarly, the greatest interspecies divergence was 15.7% between *M. acridum* and *M. robertsii*, with the least divergence between *M. guizhouense* and *M. majus* (2.5%). The overall average interspecies variation for the *Mad2* open reading frame and promoter region for all *Metarhizium* species examined was 7.4% and 9.9%, respectively. The intraspecies variation within the open reading frame was low in *M. robertsii* (0.2%) and *M. brunneum* (0.0%), and moderately higher in *M. guizhouense* (2.2%). In the promoter region, intraspecies variation was also low in *M. robertsii* (0.04%) and *M. brunneum* (0.1%), and higher in *M. guizhouense* (1.5%). The average estimated dN/dS ratio for *Mad2* was 0.31.

The length of the MAD2 proteins was conserved for isolates within the PARB clade, which includes *M. pingshaense*, *M. robertsii*, and *M. brunneum*, as well as Ontario isolates of *M. guizhouense*, with a length of 306 amino acids. ARSEF 6238 (*M. guizhouense*) had the longest MAD2 protein at 310 a.a. *M. majus* and *M. lepidiotae* have a MAD2 protein length of 305 and 307 a.a., respectively. Overall, *M. acridum* had the shortest MAD2 protein at 295 a.a., including a 12 amino acid deletion directly after the Thr-rich tandem repeats present in domain B. Analysis of the MAD2 protein sequence revealed a variable region in the N-terminal ligand binding region, with the variablilty conserved within a species.

Analysis of the *Mad2* promoter regions revealed differences in putative transcriptional elements. A stress responsive element (STRE) (AGGGG) was present twice within all species, except for *M. robertsii* and *M. acridum* isolates. *M robertsii* and *M. acridum* possessed a post-diauxic shift (PDS) element (AAGGGA) in place of the second STRE copy (upstream location -109 in *M. robertsii*). Interestingly, a degenerative TATA box (TATG) was present in the promoter of *M. robertsii* as a repeat sequence, containing five repeats (upstream location -604). *M. pingshaense* contained three repeats in this region, while *M. majus* and *M. guizhouense* contained one. *M. brunneum*, *M. acridum*, and *M. lepidiotae* all lacked a TATG repeat in this region. This degenerative TATA box was also present prior to a TATA box-like sequence (TACATA) in isolates of the PARB clade, which includes *M. pingshaense*, *M. robertsii*, and *M. brunneum* (upstream location -266 in *M. robertsii*). The TATA-box-like sequence was also present within the promoter region of the MGT isolates, which includes *M. majus* and *M. guizhouense*, although they lacked the degenerative TATA box. *M. lepidiotae* had two TATG repeats in this region, while *M. acridum* had one TATG sequence present. *M. acridum* and *M. lepidiotae* lacked the TATA-box-like sequence.

### Phylogenetic analysis of 5′ EF-1α, *Mad1*, and *Mad2*

**Figure 1:**
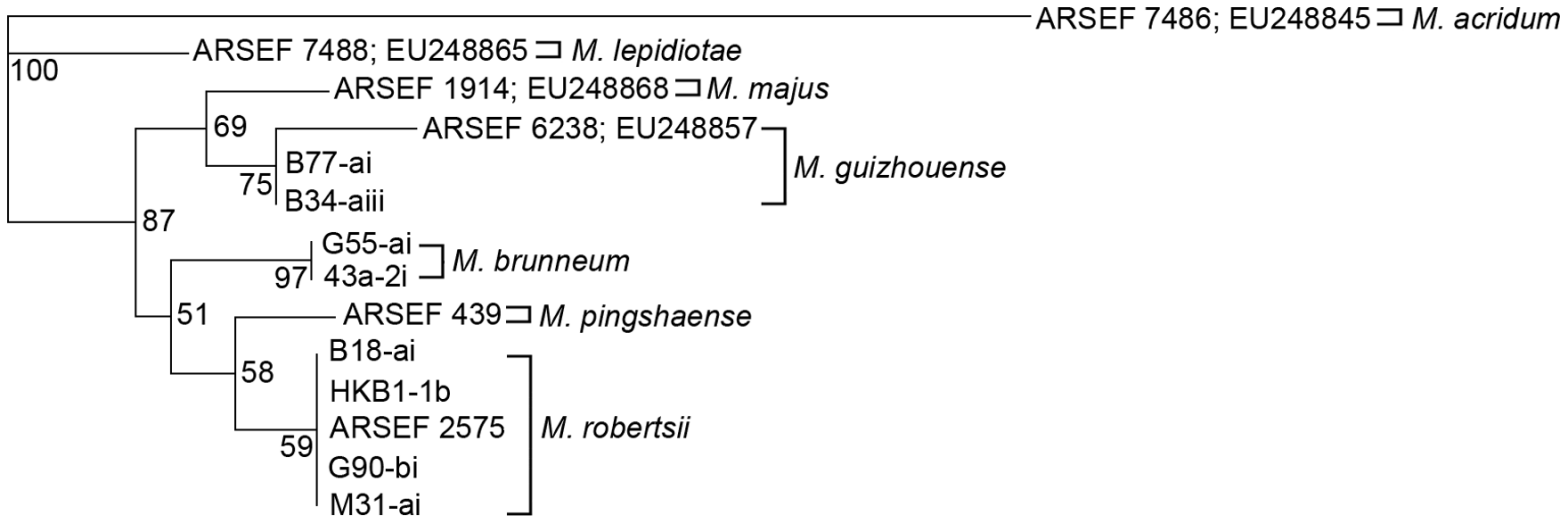
Maximum parsimony (MP) phylogenetic tree of 5′ EF-1á sequences of *Metarhizium* isolates. Bootstrap values are based on 1000 pseudoreplicates.

The 5′ EF-1α phylogenetic tree for all fourteen isolates segregated according to species, including the division of the PARB clade, which includes isolates of *M. pingshaense*, *M. robertsii*, and *M. brunneum*, and the MGT clade, which includes isolates of *M. majus*, and *M. guizhouense* (Fig. 1).

**Figure 2:**
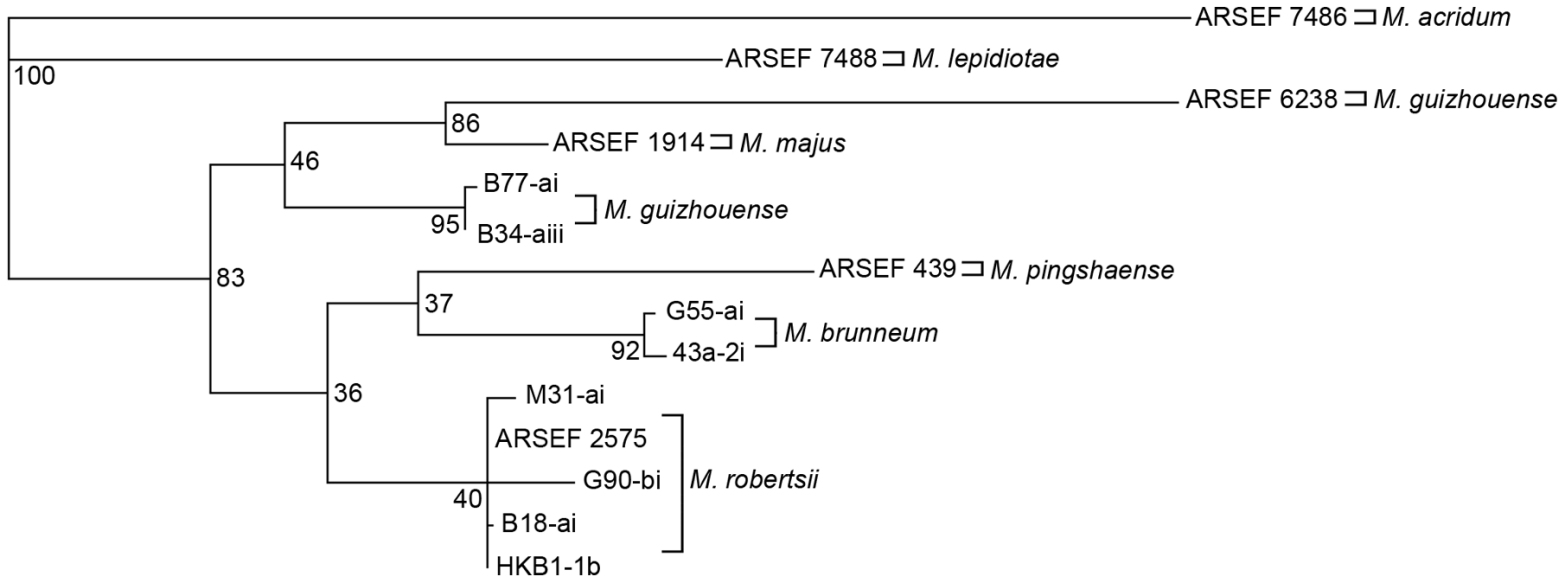
Maximum parsimony (MP) phylogenetic tree of *Mad1* full gene sequences of *Metarhizium* isolates. Bootstrap values are based on 1000 pseudoreplicates.

**Figure 3:**
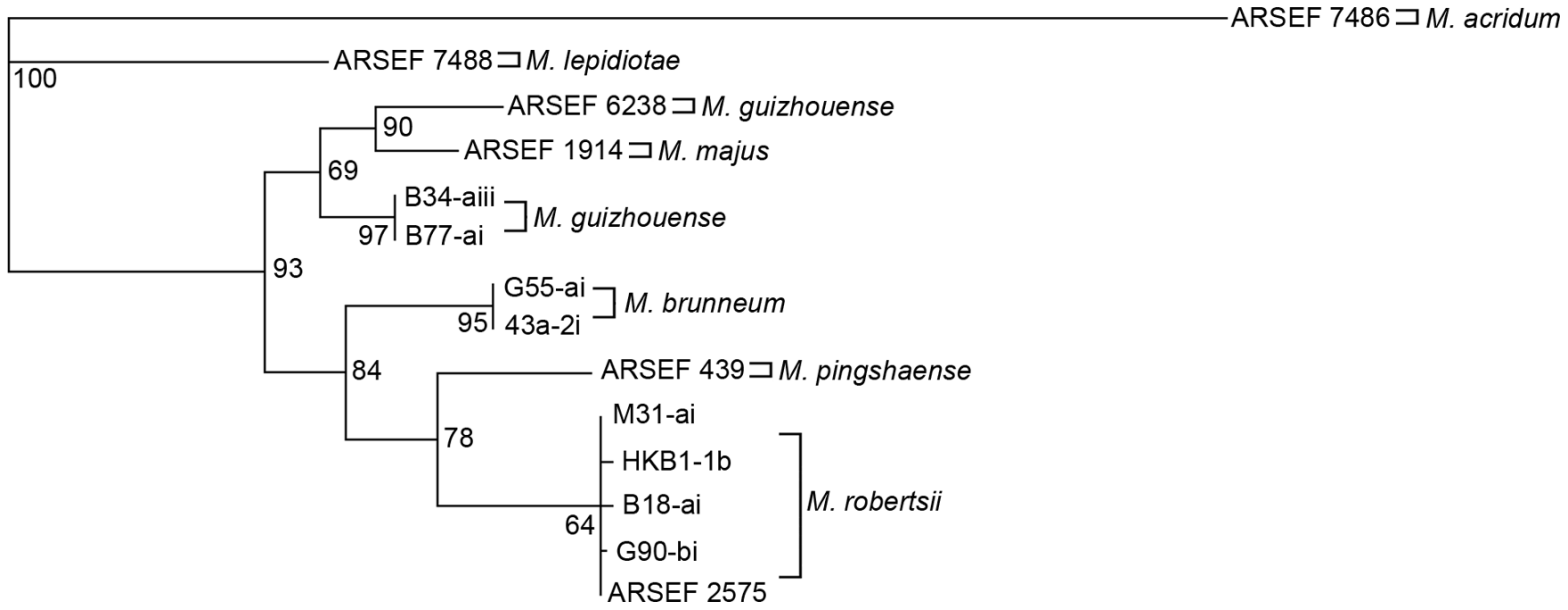
Maximum parsimony (MP) phylogenetic tree of *Mad2* full gene sequences of *Metarhizium* isolates. Bootstrap values are based on 1000 pseudoreplicates.

The phylogenetic trees for the *Mad1* and *Mad2* full gene sequences also formed divisions that were consistent with the PARB and MGT clades (Fig. 2 and 3). The PARB clade isolates all formed species-specific nodes consistent with their 5′ EF-1α identification, however, in the *Mad1* tree *M. pingshaense* and *M. brunneum* were grouped together (Fig. 2), while *M. pingshaense* and *M. robertsii* grouped together in the 5′ EF-1α and *Mad2* trees (Fig. 1 and 3). In the *Mad1* and *Mad2* phylogenetic trees, ARSEF 6238 (*M. guizhouense*) and ARSEF 1914 (*M. majus*) grouped together to form a separate node from the Ontario isolates of *M. guizhouense* within the MGT clade. Overall, the *Mad2* tree had the best resolution, with the highest bootstrap values for each node.

The congruency indices (*I*_cong_) calculated for both trees derived from the promoter regions of *Mad1* and *Mad2* in comparison to 5′ EF-1α were each 2.03 (p = 1.66×10^−6^) (Table 1). That is, the phylogenetic trees of the *Mad1* and *Mad2* promoter regions were equally congruent to the phylogenetic tree for 5′ EF-1α. The maximum agreement subtree (MAST), for 5′ EF-1α and the phylogenetic trees of the promoter regions each contained 11 terminal nodes in order for perfect congruence to occur (Table 1). The phylogenetic trees derived from the full gene DNA sequence of *Mad1*, as well as trees derived from the open reading frame DNA sequence and protein sequence were equally congruent to the 5′ EF-1α tree. In each case, the congruency index was 1.66 (p = 2.31×10^−4^). In each pairwise tree comparison the maximum agreement subtree (MAST) for 5′ EF-1α and each of the *Mad1* trees (full gene DNA sequence, open reading frame DNA sequence, and protein sequence) all contained 9 terminal nodes. For the 5′ EF-1α tree and *Mad2* trees derived from the full gene DNA sequence, open reading frame DNA sequence, and protein sequence, *I*_cong_ for each pairwise comparison was calculated to be 1.84 (p = 1.96×10^−5^). The MAST for 5′ EF-1α and the *Mad2* trees all contained 10 terminal nodes. The topology of the phylogenetic trees, as well as a higher *I*_cong_ and MAST (Table 1), indicated that the *Mad2* phylogenetic trees were more congruent with the 5′ EF-1α tree than the *Mad1* trees.

**Table 1:** Congruency values for pairwise comparisons of *Mad1* and *Mad2* phylogenetic trees to the 5′ EF-1α phylogenetic tree.

		Promoter	ORF (DNA)	ORF	Full gene**
		region* (DNA)		(Protein)	(DNA)
*Mad1*	I-cong	2.03	1.66	1.66	1.66
	p-value	1.66×10^−6^	2.31×10^−4^	2.31×10^−4^	2.31×10^−4^
	MAST	11	9	9	9
*Mad2*	I-cong	2.03	1.84	1.84	1.84
	p-value	1.66×10^−6^	1.96×10^−5^	1.96×10^−5^	1.96×10^−5^
	MAST	11	10	10	10

* ∼800 base pair DNA sequence prior to open reading frame (ORF).

** Promoter and open reading frame DNA sequences combined.

## Discussion

Here, we amplified and cloned the full *Mad1* and *Mad2* genes in fourteen isolates of seven different species of *Metarhizium* in order to assess the gene variability. *M. acridum*, the acridid-specific pathogen [16], [18], was found to have relatively more insertions and deletions within the open reading frames of *Mad1* and *Mad2*, respectively, specifically within the Thr-rich tandem repeat region in domain B of both proteins. *Mad2* variability between species was identified within putative transcriptional elements, including STRE, PDS, the degenerative TATA box, and TATA box-like regions. Additionally, phylogenetic analysis of 5′ EF-1α, *Mad1*, and *Mad2* revealed that the evolution of the *Mad2* gene was more congruent with the phylogeny of 5′ EF-1α than *Mad1*, suggesting plant host, rather than insect host, was a probable influence in the divergence among *Metarhizium* species.

In general, it was found that *Mad1* and *Mad2* were largely conserved within a species. However, intraspecies variation for *M. guizhouense* was high in comparison to *M. robertsii* and *M. brunneum*. This was especially notable for the *Mad1* open reading frame, in which variation within *M. guizhouense* was greater than the variation between *M. robertsii* and *M. brunneum*. However, Ontario isolates of *M. guizhouense* had very low intraspecies variation, similar to that of *M. robertsii* and *M. brunneum*. Additionally, ARSEF 6238 (*M. guizhouense*) formed a group with ARSEF 1914 (*M. majus*), separate from the Ontario isolates of *M. guizhouense* in all *Mad1* and *Mad2* phylogenetic trees. This influenced the incongruencies in the *Mad1* and *Mad2* phylogenies when compared to the 5′ EF-1α tree. This may be due to geographic divergence within *M. guizhouense*, since ARSEF 1914 and ARSEF 6238 were isolated in the Philippines and China, respectively [1]. Interestingly, Bischoff *et al*. [1] accepted *M. majus* and *M. guizhouense* at the species rank due to congruence between conidial size and the 5′ EF-1α phylogeny, although these species did not meet the molecular genealogical concordance criteria. However, Japanese isolates demonstrated that the conidial sizes of *M. majus* and *M. guizhouense* were incongruent with the 5′ EF-1α phylogeny [19]. This incongruence within the MGT clade warrants further investigation in order to fully resolve species ranks which may be obfuscated by population genetic differences within a *Metarhizium* species.

*M. acridum*, which is a species that displays insect host specificity, particularly pathogenic to acridids (grasshoppers and locusts) [16], [18], had the longest MAD1 protein. This includes an 11 amino acid insertion that gave the *M. acridum* MAD1 protein eight tandem repeats of GKETTPAQQTTP within domain B, as opposed to the six repeats in all other isolates. This is putatively a functional difference, as it is presumed that a higher number of repeats could increase the distance between the cell wall and the N-terminal ligand binding region [17]. Additionally, *M. acridum* possessed a variable region in the N-terminal ligand binding region, which could putatively cause a difference in adherence. However, when the *Mad1* gene from *M. acridum* was inserted into *M. robertsii*, there was no difference in cuticle adhesion or virulence (St. Leger, pers. comm.). Conversely, *M. acridum* had the shortest MAD2 protein, including a 12 amino acid deletion directly after the Thr-rich repeats in domain B. This may also have a functional implication that may limit its ability to associate with plants. Phylogenetic analysis of 5′ EF-1α, *Mad1*, and *Mad2* also shows this species is highly divergent from other *Metarhizium* species.

Within the MAD2 protein sequence, a variable region was readily identified within the N-terminal ligand binding region. Interestingly, the variability was conserved within a species. This amino-terminal domain has been implicated in adhesive interactions in the ALS proteins of *C. albicans* [20], which are similar to the MAD1 and MAD2 proteins [17]. It may be possible that this variability causes differences in adhesion to various plants among species of *Metarhizium*.

Overall, genetic variation was slightly greater in the *Mad2* open reading frame (7.4%) in comparison to *Mad1* (6.7%), but noticeably higher in the *Mad2* promoter region (9.9%) in comparison to the *Mad1* promoter (7.1%). Analysis of the *Mad1* promoter did not identify any variable transcriptional elements. Future research could focus on the expression of *Mad2* between species since there was variation present within the promoter region. Several putative transcriptional elements have been identified within the *Mad2* promoter [21], however, the analyses presented here focused on the variable STRE, PDS, degenerative TATA box, and TATA box-like regions. The stress response element (STRE) activates genes under various stress conditions, including glucose starvation [22], [23]. Similarly, the post diauxic shift (PDS) element mediates transcriptional activation in response to nutritional limitation [24], [25]. The presence of these transcriptional elements is consistent with the finding that *Mad2* is upregulated under nutrient deprivation [21].

Interestingly, it has been found that the expression of cell wall and stress response genes evolved at an accelerated rate following the transfer of *M. robertsii* from a semitropical to a temperate soil community [26]. It was also found that cell wall genes with significantly altered expression were enriched for TATA boxes. Conversely, virulence determinants were unaltered [26]. *M. robertsii*, which has demonstrated a more generalist ability to colonize plant rhizosphere when compared to *M. brunneum* and *M. guizhouense* [10], contained the most TATG repeats within the degenerative TATA box region. It also contains a TATG repeat prior to the TATA box-like sequence, which the other species lack. Whether this contributes to the generalist nature of the plant association is unknown. Also, the length of the MAD2 protein is conserved within the PARB clade, including the Ontario isolates of *M. guizhouense*. This is notable, since Ontario isolates of *M. robertsii*, *M. brunneum*, and *M. guizhouense* have shown plant rhizosphere associations [10].

Overall, variation within the DNA and protein sequences of the *Mad1* and *Mad2* genes, were largely species-specific. This is expected, as these genes would have diverged during speciation. However, the higher amount of variation, especially in the promoter region, suggests *Mad2* had diverged more than *Mad1*, and phylogenetic analysis indicated that *Mad2* is more congruent with the 5′ EF-1α phylogeny, which is used for species identification [1]. Also, variation within the TATA box-like region of the *Mad2* promoter was conserved within a clade. This would suggest that in evolutionary terms, *Mad2*, the plant adhesin, has diverged among *Metarhizium* lineages, contributing to clade- and species-specific variation. Conversely, it appears that *Mad1* has been largely conserved. This is reflected in the average estimated dN/dS ratio, which is higher in *Mad2* (0.31) than in *Mad1* (0.20), suggesting that there is more stabilizing selection for *Mad1*, as there is a higher relative abundance of nonsynonymous mutations.

One explanation for the results observed is that the stabilizing selection for *Mad1* has reduced variation and caused incongruency with 5′ EF-1α. The promoter regions are both equally congruent to 5′ EF-1α. While EF-1α is highly conserved [27], the 5′ region used in these analyses contains a large portion of intronic nucleotides (>60% when aligned with GenBank Accession AAR16425). As such, the promoter regions of the *Mad* genes and the intronic regions of 5′ EF-1α would both accumulate random substitutions during evolution. *Mad2*, which has demonstrated a degree of stabilizing selection, would have fewer accumulated random mutations. Lastly, *Mad1*, which has shown more stabilizing selection and less variation than *Mad2*, would have accumulated even fewer random mutations, resulting in more incongruency with the 5′ EF-1α phylogenetic tree. Previous studies on insect infection related genes (i.e. *Pr*1 and *Ntl*) have also demonstrated a high degree of stabilizing selection [6], [28].

There is evidence that plant host association may play an important role in the evolutionary divergence within the genus *Metarhizium* with the exception of the acridid-specific *M. acridum* and possibly *M. majus*, which has demonstrated specificity for Coleopteran insects, particularly scarabs [18], [29], [30]. Ontario species of *Metarhizium* have shown plant rhizosphere specificity [10] and *M. robertsii* is an endophyte [14]. Whole genome analyses have also suggested that the genus *Metarhizium* evolved from endophytes or plant pathogens [16].

While the *Mad2* plant adhesin gene showed a higher amount of variability than *Mad1*, and was more congruent with 5′ EF-1α, it is difficult to ascertain whether this is due to plant relationships alone. Over the course of time, a number of factors may have contributed differentially to the evolution of *Metarhizium* species. The genetic differences found may be the residual effects derived from an ancestral plant-associated relative. While phylogenetic evidence suggests that plant interactions have had the greater role in shaping the evolution of this fungal genera, it is possible that insect associations may have been influential on the more recent evolution of *Metarhizium*. While other abiotic and biotic factors cannot be excluded in contributing to species divergences, it appears that plant relationships have been a driving factor in the evolution of *Metarhizium* species.

## Methods

### *Metarhizium* isolates

Fourteen isolates of *Metarhizium*, representing seven of nine *Metarhizium spp.* complex species identified by Bischoff *et al*. [1], were used in this study. Eight isolates were obtained from soil and plant root samples from various locations in Ontario, Canada [9], [10]; *M. robertsii* isolates HKB1-1b, B18-ai, M31-ai, and G90-ai; *M. brunneum* isolates 43a-2i, and G55-ai; and *M. guizhouense* isolates B34-aiii, and B77-ai. *Metarhizium* isolates representing six species were obtained from the USDA-ARSEF (Ithaca, New York); ARSEF 6238 (*M. guizhouense*), ARSEF 439 (*M. pingshaense*) and ex-type isolates ARSEF 7488 (*M. lepidiotae*), ARSEF 7486 (*M. acridum*), ARSEF 2575 (*M. robertsii*), and ARSEF 1914 (*M. majus*) [1]. Isolates were grown on potato dextrose agar (Difco) plates at 27°C for 10 days in order to obtain conidia.

### DNA extraction

Conidia were inoculated into 50 mL 0.2% (w/v) yeast extract, 1% peptone, 2% dextrose (YPD) broth in flasks. The flasks were incubated at 27°C and shaken at 200 rpm for 3 to 4 days, until sufficient mycelia had accumulated. The mycelia were removed by vacuum filtration onto Fisherbrand® P8 filter paper, washed with distilled water, and crushed in liquid nitrogen using a mortar and pestle. DNA was extracted using the DNeasy Plant Mini Kit (QIAGEN). Extracted DNA was quantified using a NanoVue spectrophotometer (GE).

### PCR, primer walking, gene cloning, and sequencing

Primers were designed by using the sequences of the *Metarhizium* adhesin-like protein 1 (*Mad1*), and *Metarhizium* adhesin-like protein 2 (*Mad2*) derived from ARSEF 2575 [17], and deposited in GenBank (accession No. DQ338437 and DQ338439, respectively). The upstream sequence for *Mad1* was obtained using Y-shaped adaptor dependent extension (YADE) [31].

PCR amplifications were performed in a total volume of 50 µL, which included 5 µL 10X Standard PCR Buffer (NEB), 1 µL dNTPs (10 mM each dATP, dCTP, dGTP, dTTP) (QIAGEN), 10 pmol each of the opposing amplification primers (Sigma), 0.5 µL *Taq* polymerase (NEB), and 500ng genomic DNA. The following PCR conditions were used for *Mad1* amplification: initial denaturation, 1 minute at 94°C, then 30 cycles of denaturation, 1 minute at 94°C; annealing, 1 minute at 60°C; extension, 4.5 minutes at 72°C; and final extension, 10 minutes at 72°C. The same PCR conditions were used for *Mad2* amplification, with an annealing temperature of 56°C and an extension time of 3 minutes. Table 2 lists the primers used to amplify all *Mad1* and *Mad2* sequences.

**Table 2:** Primers used to amplify all *Mad1* and *Mad2* sequences in *Metarhizium* isolates.

Gene: Isolate(s)	Primer Sequence (5′–3′)
*Mad1*: All Isolates	(F) GCT TGT GCC CTG TGT TCC
	(R) AAG ATT ACA GAA TGC CAG CCC T
*Mad2*: A2575, HKB1-1b, B18-ai, M31-ai,	(F) GCG GCT AAT TTT TGA CTA C
G90-bi, 43a-2i, G55-ai, A7488, A7486	(R) TCA TAG CAC AAA TGA GTT GTA T
*Mad2*: A439	(F) GGA TAT TCA GTC GTG GCT
	(R) TCA TAG CAC AAA TGA GTT GTA
*Mad2*: A6238, A1914	(F) GCT TGC TCG TTA GAC ACA
	(R) TTA GTG TCG GAG GAA TAG AT
*Mad2*: B34-aiii, B77-ai	(F) AGT GAC TTG GTG GGA TAA G
	(R) TTA GTG TCG GAG GAA TAG AT

The 5′ region of the translation elongation factor 1-alpha (EF-1α) gene was amplified according to previously described conditions [1], [32], [33] for isolates ARSEF 2575 (*M. robertsii*) and ARSEF 439 (*M. pingshaense*).

Full DNA sequences for *Mad1* and *Mad2* were obtained for all isolates by primer walking (DNA Walking Speedup kit; Seegene). Amplified PCR products were separated by gel electrophoresis, excised, and purified with a QIAquick gel extraction kit (QIAGEN). Purified PCR products were cloned using pGEM-T Easy, as per manufacturer's instructions (Promega). Plasmid DNA was extracted using a GenElute Plasmid Miniprep Kit (Sigma), and inserts were sequenced using vector sequencing primers (SP6 and T7) by the Core Molecular Biology Facility at York University (Toronto, Canada).

The 5′ EF-1α sequences were obtained from GenBank for isolates HKB1-1b (HM748301), B18-ai (HM748302), M31-ai (HM748303), G90-bi (HM748304), 43a-2i (FJ229493), G55-ai (HM748305), B77-ai (HM748307), B34-aiii (HM748308), ARSEF 7488 (EU248865), ARSEF 7486 (EU248845), ARSEF 6238 (EU248857), and ARSEF 1914 (EU248868). GenBank accession numbers for the *Mad* genes and 5′ EF-1α sequenced for this study were: HKB1-1b (*Mad1*: KC484637; *Mad2*: KC484624), B18-ai (*Mad1*: KC484638; *Mad2*: KC484625), M31-ai (*Mad1*: KC484639; *Mad2*: KC484626), G90-bi (*Mad1*: KC484640; *Mad2*: KC484627), 43a-2i (*Mad1*: KC484642; *Mad2*: KC484629), G55-ai (*Mad1*: KC484643; *Mad2*: KC484630), B34-aiii (*Mad1*: KC484644; *Mad2*: KC484631), B77-ai (*Mad1*: KC484645; *Mad2*: KC484632), ARSEF 7488 (*Mad1*: KC484648; *Mad2*: KC484635), ARSEF 7486 (*Mad1*: KC484649; *Mad2*: KC484636), ARSEF 6238 (*Mad1*: KC484646; *Mad2*: KC484633), ARSEF 2575 (5′ EF-1α: KC484650), ARSEF 1914 (*Mad1*: KC484647; *Mad2*: KC484634) and ARSEF 439 (*Mad1*: KC484641; *Mad2*: KC484628; 5′ EF-1α: KC484651).

### Sequence and phylogenetic analysis

DNA sequences were aligned using Clustal_X 2.1 [34] using the default settings. Translated protein sequences were identified through a multiple sequence alignment with protein sequences derived from ARSEF 2575 [17], for MAD1 and MAD2 (accession No. ABC65821 and ABC65823, respectively). Pairwise comparisons were carried out using EMBOSS Needle [34], [35]. The nonsynonymous/synonymous rate ratio (dN/dS) was estimated using the ETH Codon Suite, which uses an empirical codon substitution matrix [36], and estimates dN and dS according to Nei and Gojobori [37].

Molecular phylogenetic analysis of the 5′ EF-1α, *Mad1* and *Mad2* sequences was conducted in order to evaluate the phylogenetic relationship of the genes. Maximum parsimony (MP) phylogenetic trees were constructed using PHYLIP 3.69 [38], as previously described [10]. Nonparametric bootstrapping was conducted using 1000 pseudoreplicates, with 10 random addition replicates per parsimony run, and subtree pruning and regrafting (SPR) branch swapping. The congruency index (*I*_cong_) and maximum agreement subtree (MAST) were calculated using the *I*_cong_ online tool [39], which calculates the MAST values following Berry and Nicolas [40].

## References

[1] Bischoff JF, Rehner SA, Humber RA (2009) A multilocus phylogeny of the *Metarhizium anisopliae* lineage. Mycologia 101: 512–530. 19623931 10.3852/07-202

[2] Bridge PD, Williams MAJ, Prior C, Paterson RRM (1993) Morphological, biochemical and molecular characteristics of *Metarhizium anisopliae* and *M. flavoviride* . J Gen Microbiol 139: 1163–1169.

[3] Bridge PD, Prior C, Sagbohan J, Lomer CJ, Carey M, et al (1997) Molecular characterization of isolates of *Metarhizium* from locusts and grasshoppers. Biodiversity Conserv 6: 177–189.

[4] Fegan M, Manners JM, Maclean DJ, Irwin JAG, Samuels KDZ, et al (1993) Random amplified polymorphic DNA markers reveal a high degree of genetic diversity in the entomopathogenic fungus *Metarhizium anisopliae* var. *anisopliae* . J Gen Microbiol 139: 2075–2081. 8245834 10.1099/00221287-139-9-2075

[5] Leal SCM, Bertioli DJ, Butt TM, Peberdy JF (1994) Characterization of isolates of the entomopathogenic fungus *Metarhizium anisopliae* by RAPD-PCR. Mycol Res 98: 1077–1081.

[6] Leal SCM, Bertioli DJ, Butt TM, Carder JH, Burrows PR, et al (1997) Amplification and restriction endonuclease digestion of the Pr1 gene for the detection and characterization of *Metarhizium* strains. Mycol Res 101: 257–265.

[7] Riba G, Bouvier-Fourcade I, Caudal A (1986) Isoenzymes polymorphism in *Metarhizium anisopliae* (Deuteromycotina: Hyphomycetes) entomogenous fungi. Mycopathology 96: 161–169.

[8] Tigano-Milani MS, Gomes ACMM, Sobral BWS (1995) Genetic variability among Brazilian isolates of the entomopathogenic fungus *Metarhizium anisopliae* . J Invertebr Pathol 65: 206–210.

[9] Bidochka MJ, Kamp AM, Lavender TM, de Koning J, de Croos JNA (2001) Habitat association of two genetic groups of the insect-pathogenic fungus *Metarhizium anisopliae*: uncovering cryptic species? Appl Environ Microbiol 67: 1335–1342. 11229929 10.1128/AEM.67.3.1335-1342.2001 PMC92732

[10] Wyrebek M, Huber C, Sasan RK, Bidochka MJ (2011) Three sympatrically occurring species of *Metarhizium* show plant rhizosphere specificity. Microbiology 157: 2904–2911. 21778205 10.1099/mic.0.051102-0

[11] Bruck DJ (2005) Ecology of *Metarhizium anisopliae* in soilless potting media and the rhizosphere: implications for pest management. Biol Control 32: 155–163.

[12] Bruck DJ (2010) Fungal entomopathogens in the rhizosphere. BioControl 55: 103–112.

[13] Hu G, St. Leger RJ (2002) Field studies using a recombinant mycoinsecticide (*Metarhizium anisopliae*) reveal that it is rhizosphere competent. Appl Environ Microbiol 68: 6383–6387. 12450863 10.1128/AEM.68.12.6383-6387.2002 PMC134390

[14] Sasan RK, Bidochka MJ (2012) The insect-pathogenic fungus *Metarhizium robertsii* (Clavicipitaceae) is also an endophyte that stimulates plant root development. Am J Bot 99: 101–107. 22174335 10.3732/ajb.1100136

[15] Spatafora JW, Sung GH, Sung HM, Hywel-Jones L, White JF (2007) Phylogenetic evidence for an animal pathogen origin of ergot and the grass endophytes. Mol Ecol 16: 1701–1711. 17402984 10.1111/j.1365-294X.2007.03225.x

[16] Gao Q, Jin K, Ying SH, Zhang Y, Xiao G, et al (2011) Genome sequencing and comparative transcriptomics of the model entomopathogenic fungi *Metarhizium anisopliae* and *M. acridum* . PLoS Genet 7: e1001264. 21253567 10.1371/journal.pgen.1001264 PMC3017113

[17] Wang C, St. Leger RJ (2007) The MAD1 adhesin of *Metarhizium anisopliae* links adhesion with blastospore production and virulence to insects and the MAD2 adhesin enables attachment to plants. Eukaryot Cell 6: 808–816. 17337634 10.1128/EC.00409-06 PMC1899246

[18] Wang S, Fang W, Wang CS, St. Leger RJ (2011) Insertion of an esterase gene into a specific locust pathogen (*Metarhizium acridum*) enables it to infect caterpillars. PLoS Pathog 7: e1002097. 21731492 10.1371/journal.ppat.1002097 PMC3121873

[19] Nishi O, Hasegawa K, Iiyama K, Yasunaga-Aoki C, Shimizu S (2011) Phylogenetic analysis of *Metarhizium* spp. isolated from soil in Japan. Appl Entomol Zool 46: 301–309.

[20] Hoyer LL (2001) The *ALS* gene family of *Candida albicans* . Trends Microbiol 9: 176–180. 11286882 10.1016/s0966-842x(01)01984-9

[21] Barelli L, Padilla-Guerrero IE, Bidochka MJ (2011) Differential expression of insect and plant specific adhesin genes, *Mad1* and *Mad2,* in *Metarhizium robertsii* . Fungal Biol 115: 1174–1185. 22036295 10.1016/j.funbio.2011.08.003

[22] Görner W, Durchschlag E, Martinez-Pastor MT, Estruch F, Ammerer G, et al (1998) Nuclear localization of the C2H2 zinc finger protein Msn2p is regulated by stress and protein kinase A activity. Gene Dev 12: 586–597. 9472026 10.1101/gad.12.4.586 PMC316529

[23] Marchler G, Schuller C, Adam G, Ruis H (1993) A *Saccharomyces cerevisiae* UAS element controlled by protein kinase A activates transcription in response to a variety of stress conditions. EMBO J 12: 1997–2003. 8387917 10.1002/j.1460-2075.1993.tb05849.x PMC413422

[24] Boorstein WR, Craig EA (1990) Regulation of a yeast HSP70 gene by a cAMP responsive transcriptional control element. EMBO J 9: 2543–2553. 1695149 10.1002/j.1460-2075.1990.tb07435.x PMC552285

[25] Pedruzzi I, Burckert N, Egger P, De Virgilio C (2000) *Saccharomyces cerevisiae* Ras/cAMP pathway controls post-diauxic shift element-dependent transcription through the zinc finger protein Gis1. EMBO J 19: 2569–2579. 10835355 10.1093/emboj/19.11.2569 PMC212766

[26] Wang S, O'Brien TR, Pava-Ripoll M, St. Leger RJ (2011) Local adaptation of an introduced transgenic insect fungal pathogen due to new beneficial mutations. PNAS 108: 20449–20454. 22143757 10.1073/pnas.1113824108 PMC3251136

[27] Nakazato L, Dutra V, Broetto L, Staats CC, Vainstein MH, et al (2006) Development of an expression vector for *Metarhizium anisopliae* based on the *tef-1α* homologous promoter. Appl Microbiol Biotechnol 72: 521–528. 16402168 10.1007/s00253-005-0292-3

[28] Small CL, Donaldson N, Bidochka MJ (2004) Nucleotide sequence variation does not relate to differences in kinetic properties of neutral trehalase from the insect-pathogenic fungus *Metarhizium anisopliae* . Curr Microbiol 48: 428–434. 15170238 10.1007/s00284-003-4228-9

[29] Lin KJ, Roberts DW (1986) The production of destruxin by the entomogenous fungus *Metarhizium anisopliae* var. *majus* . J Invertebr Pathol 47: 120–122.

[30] Wang B, Kang Q, Lu Y, Bai L, Wang C (2012) Unveiling the biosynthetic puzzle of destruxins in *Metarhizium* species. PNAS 109: 1287–1292. 22232661 10.1073/pnas.1115983109 PMC3268274

[31] Fang W, Leng B, Xiao Y, Jin K, Ma J, et al (2005) Cloning of *Beauveria bassiana* chitinase gene *Bbchit1* and its application to improve fungal strain virulence. Appl Environ Microbiol 71: 363–370. 15640210 10.1128/AEM.71.1.363-370.2005 PMC544255

[32] Rehner SA, Buckley E (2005) A *Beauveria* phylogeny inferred from nuclear ITS and EF1-a sequences: evidence for cryptic diversification and links to *Cordyceps* teleomorphs. Mycologia 97: 84–98. 16389960 10.3852/mycologia.97.1.84

[33] Bischoff JF, Rehner SA, Humber RA (2006) *Metarhizium frigidum* sp. nov.: a cryptic species of *M. anisopliae* and a member of the *M. flavoviride* complex. Mycologia 98: 737–735. 17256577 10.3852/mycologia.98.5.737

[34] Larkin MA, Blackshields G, Brown NP, Chenna R, McGettigan PA, et al (2007) Clustal W and Clustal X version 2.0. Bioinformatics 23: 2947–2948. 17846036 10.1093/bioinformatics/btm404

[35] Rice P, Longden I, Bleasby A (2000) EMBOSS: The European Molecular Biology Open Software Suite. Trends Genet 16: 276–277. 10827456 10.1016/s0168-9525(00)02024-2

[36] Schneider A, Cannarozzi GM, Gonnet GH (2005) Empirical codon substitution matrix. BMC Bioinformatics 6: 134–140. 15927081 10.1186/1471-2105-6-134 PMC1173088

[37] Nei M, Gojobori T (1986) Simple methods for estimating the numbers of synonymous and nonsynonymous nucleotide substitutions. Mol Biol Evol 3: 418–426. 3444411 10.1093/oxfordjournals.molbev.a040410

[38] Felsenstein J (1989) PHYLIP (phylogeny inference package). Version 3.2. Cladistics 5: 164–166.

[39] De Vienne DM, Giraud T, Martin OC (2007) A congruence index for testing topological similarity between trees. Bioinformatics 23: 3119–3124. 17933852 10.1093/bioinformatics/btm500

[40] Berry V, Nicolas F (2006) Improved parameterized complexity of the maximum agreement subtree and maximum compatible tree problems. IEEE/ACM Trans Comput Biol Bioinform 3: 289–302. 17048466 10.1109/TCBB.2006.39

